# The reliability and validity of the Temptations to Try Smoking Scale in a group of Chinese adolescents

**DOI:** 10.1186/s41155-023-00271-1

**Published:** 2023-10-26

**Authors:** Weishi Xie, Linya Wang, Difei Liu

**Affiliations:** 1 Department of Psychology, Key Laboratory of Philosophy and Social Science of Anhui Province On Adolescent Mental Health and Crisis Intelligence Intervention, Hefei Normal University, Hefei, Anhui Province China; 2 Anhui Mental Health Education and Guidance Center for Students, Hefei Normal University, Hefei, 230601 Anhui Province China; 3 Department of Education, Hefei University, Hefei, Anhui Province China

**Keywords:** Temptations to Try Smoking Scale (TTSS), Reliability, Validity, Adolescents

## Abstract

**Objective:**

To provide a scientifc tool, the Temptations to Try Smoking Scale (TTSS) is introduced to evaluate its reliability and validity in preventing and intervening Chinese adolescents from smoking temptations.

**Methods:**

A questionnaire, including the TTSS, the Chinese version of the Decisional Balance Scale (CDBS), the Adolescent Smoking Curiosity Scale (ASCOS), and the Sensation-Seeking Scale (SSS), is used to test 1195 Chinese adolescent volunteers (214 of them are retested after 1 month). If all six items in the TTSS are retained, the exploratory factor analysis (EFA) reveals that the TTSS exhibits a structure of two factors: positive social and curiosity/stress.

**Results:**

The confrmatory factor analysis (CFA) shows that the two-factor model of the TTSS has the ftting indices *χ*^2^/*df* = 2.35, *RMSEA* = 0.06, and *CFI* = 0.99, which are better than those of its single-factor model. The total scores of the TTSS, positive social, and curiosity/stress are positively correlated with the scores of Pros, ASCOS, TAS, and Dis of SSS but negatively correlated with the Cons, hereby exhibiting good criterion-related validity. The internal consistency coefcient of the TTSS is 0.89, and the retest reliability is 0.90.

**Conclusion:**

Therefore, the TTSS has good reliability and validity for Chinese adolescents and can be used as an efective tool to evaluate adolescents’ smoking temptations in China.

## Introduction

The harm of tobacco is an urgent public health problem to be solved. According to the survey results of the World Health Organization (2020), 80,000 to 100,000 adolescents try smoking every day, and nearly half of them come from Asia. Moreover, the number of adolescent smokers increases gradually, and first-time smoking begins at a younger age (Pan et al., 2020). According to the *Chinese health hazards of smoking in 2020* (National Health Commission of the People’s Republic of China, 2020), more than 300 million people in China smoke, with 26.6% of them aged 15 or above. It is estimated that the number of smokers will increase by 2 million per year in 2030 and 3 million per year in 2050. Furthermore, the youth tobacco epidemic in 2011 showed that 22.5% of middle school students had tried smoking. Among them, adolescents aged between 12 and 14, in particular, have the highest smoking rate of 26.2% (Chinese Association on Tobacco Control, 2011). The latest tobacco epidemic surveillance report, released by the Chinese Center for Disease Control and Prevention (2021), also showed that 16.7% of Chinese adolescents had tried smoking in 2021, indicating the serious problem of adolescent smoking. Due to the increasing prevalence of smoking among adolescents, the need is urgent to develop intervention methods that are aimed at preventing adolescents from smoking.

The transtheoretical model (TTM), as an integrative model of behavior change, has been widely applied to the population-based prevention interventions for unhealthy behaviors (such as addiction) and the promotion for positive behaviors (Hashemzadeh et al., 2019; Velicer et al., 2000). The TTM contains four key theoretical constructs: stages of change, processes of change, decisional balance, and situational temptations. The situational temptations, adapted from Bandura’s self-efficacy theory (Bandura, 1977), are the converse of self-efficacy. The situational temptations reflect the desire and motivation of an individual to engage in a specific behavior across a series of difficult situations (Velicer et al., 2000). The smoking temptation situation reflects the strong desire to smoke (Velicer et al., 1990). Thus, the temptation situation is a good predictor of evaluating adolescents’ attempted smoking behavior (Hoeppner et al., 2012; Pallonen et al., 1998).

In 2012, McGee et al. developed the Temptations to Try Smoking Scale (TTSS) based on the TTM. The TTSS is a 6-item and 5-point scale, which aims to evaluate the situational temptations of smoking attempts from two dimensions: positive social and curiosity/stress. The higher score on the TTSS means the larger possibility for an adolescent to smoke. The TTSS scale has been used in the prevention and intervention of smoking behavior among adolescents in America, showing good psychometric characteristics (Ford et al., 2013; McGee et al., 2012; Sillice et al., 2017). Furthermore, such TTSS has also been applied in studies about adolescent smoking in England (Babbin et al., 2015). However, TTSS has not been applied in Chinese adolescents because the Chinese cultural environment is different from Western countries. Due to the serious consequences of adolescent smoking and the importance of prevention and intervention work, it is important to introduce the TTSS into promoting the healthy growth of Chinese adolescents. Thus, through the TTSS, we can explore the predictors of adolescents’ temptations to try smoking and then take related actions to reduce their temptations and further formulate the standard policies of prevention and intervention. Therefore, this study aims to use the TTSS in China and examine its reliability and validity in evaluating smoking problems for Chinese adolescents, which has been permitted by the authors of this TTSS.

According to the TTM, in the process of behavior change, the temptation to try smoking is closely related to sensation seeking and decisional balance of smoking (Prochaska & Velicer, 1997; Velicer et al., 2000). Firstly, the decisional balance of smoking is an important factor for adolescents to try smoking. Adolescents who think smoking is beneficial are more easily influenced by the temptation of smoking than those who think smoking is harmful (Plummer et al., 2001). Similar conclusions have been achieved in China (Xie et al., 2022). In addition, the temptation to try smoking also has a tight association with sensation seeking. Compared with low sensation seeking, adolescents with high sensation are more likely to seek new and intense stimulation, thus resulting in more smoking-related risk behaviors (Crawford et al., 2003). Studies in China have also reported that the decisional balance of smoking and sensation seeking has significant predictive effects on adolescents’ smoking attempts (Xie et al., 2022). Sensation seeking among adolescents is not only a risk factor for tobacco and alcohol use (Ye et al., 2011) but also a positive predictor of tobacco and alcohol use (Yuan et al., 2016). Moreover, smoking curiosity is also another notable factor to induce teenagers to smoke (Khalil et al., 2018; Xie et al., 2020). Therefore, in this study, the Decisional Balance Scale (CDBS), the Adolescent Smoking Curiosity Scale (ASCOS), and the Sensation-Seeking Scale (SSS) are also used as criterion tools to further evaluate the criterion-related validity of the TTSS in China.

In this work, the TTSS is applied in China by establishing the psychometric characteristics of the Chinese TTSS, which will work as scientific and effective measurement tools for the follow-up prevention and intervention of adolescent smoking behavior in China.

## Methods

### Participants

According to the rules, the target sample size for CFA should be more than 10 times the number of items (Nunnally & Bernstein, 1994), so the minimum sample size required for this study is 60 subjects, and Sample 1 intended for CFA meets this goal. The target sample size for correlation analysis is determined by G*Power before data collection (Faul et al., 2009). To meet the requirements of medium effect size *ρ* = 0.30 and power = 0.95 for correlation analysis, the minimum number of the required samples is 138 subjects. Both two samples (Sample 1 with 603 subjects, Sample 2 with 592 subjects) in this study meet this goal.

We have recruited subjects through a convenient sampling method. All the volunteers are middle school students from different provinces (Anhui, Henan, Hubei, and Jiangsu) in China. The sample is distributed by grade: grade 1 = 14.1%, grade 2 = 14.6%, grade 3 = 20.3%, senior 1 = 20.0%, senior 2 = 15.5%, and senior 3 = 15.5%. The survey was conducted in two phases, with Sample 1 and Sample 2 conducted in September 2019. Sample 1 contains 603 students (298 males and 305 females), who are aged between 11 and 19 (*M* = 15.12; *SD* = 2.26). Among them, 473 students have no smoking experience, 38 are smokers, and 92 have tried smoking. Sample 2 contains 592 students (289 males and 303 females) with their ages between 11 and 19 (*M* = 15.20; *SD* = 2.31), where 456 do not smoke, 32 are smokers, and 104 have attempted smoking. After 1 month (i.e., October 2019), 214 students (106 males and 108 females) with their ages between 11 and 19 (*M* = 15.22; *SD* = 2.17) in Sample 2 were selected randomly for retesting.

### Procedure

The current study has been approved by the Research Ethics Committee of Hefei Normal University. All participants read and agreed to the informed consent form before the survey, which stated the importance, objectives, voluntariness, and confidentiality principles of the survey. Before the volunteers fill out the paper questionnaires in their class, they are informed that they have the right to withdraw at any time and need to respond to all items in the questionnaire; thus, no data loss in this study. All the responses are analyzed in a de-identified manner.

### Measurements

#### The Temptations to Try Smoking

The Temptations to Try Smoking is used by following several steps: First, two psychology teachers with doctoral degrees independently translated these 6 items of TTSS into Chinese. Then, another two English teachers independently retranslated the same 6 items into Chinese again. Finally, the Chinese version of the TTSS was formed after detailed discussions by three psychological experts. The resulting Chinese TTSS is a 6-item 5-point scale (1 for “not at all tempted”; 5 for “extremely interested”), including two dimensions: positive social and curiosity/stress. The higher the score, the more likely the subjects of adolescents are to be tempted to smoke.

#### Chinese version of the Decisional Balance Scale (CDBS)

The 12-item CDBS (Chen et al., 2006a, 2006b) consists of the Pros and Cons subscales. The Cronbach’s *α* of the two subscales in the original study are 0.87 and 0.91, respectively. Responses are scored on a 5-point Likert scale (1 for “strongly disagree,” 5 for “strongly agree”). A higher score on the Pros indicates that “the volunteers think smoking brings more benefits,” while a higher score on the cons indicates more harm from smoking. In this study, Cronbach’s *α* of the two subscales are 0.88 and 0.85, respectively.

#### Adolescent Smoking Curiosity Scale (ASCOS)

The single-dimension structure Adolescent Smoking Curiosity Scale (Xie et al., 2020) includes 7 items, which is designed to examine subjects’ curiosity about smoking in three aspects: tobacco products, feelings about smoking, and the social influence of smoking. The scale is scored with a 5-point Likert scale (1 for “not at all,” 5 for “very much”), where the higher score indicates the higher levels of curiosity about smoking. In this study, Cronbach’s *α* was 0.91, which is nearly the same as 0.92 of the original study.

#### Sensation-Seeking Scale (SSS)

The Sensation-Seeking Scale (Chen et al., 2006a, 2006b) contains 30 items in two factors: TAS (15 items) and Dis (15 items). The internal consistency coefficient of the scale is 0.86, and the reliability of the two factors are 0.79 and 0.87, respectively. The 3-point Likert scale is used, where the volunteers are asked to respond by following the strategy: 0 for “Do not want to do,” 1 for “Want to do, but not necessarily do,” and 2 for “If I have the chance, I will do it.” A higher score indicates a higher level of sensation seeking. The Cronbach’s *α* of the scale in this study is 0.85, and the two factors are 0.78 and 0.84, respectively.

### Statistical analysis

The data analysis is carried out in two phases. In phase 1, to obtain the quality of each item, SPSS 24.0 is first used for item analysis on Sample 1. Then, the exploratory factor analysis (EFA) is used to explore the structure of factors, as well as examine the factor loading of each item. Based on the above results, one determines whether any item needs to be deleted. In phase 2, AMOS 24.0 is used to conduct confirmatory factor analysis (CFA) on Sample 2 so that we can verify the structure of the factor. Then, to determine the correlation between TTSS and the criterion variable, Pearson correlation in SPSS 24.0 is used. Finally, by using SPSS 24.0, the reliability of TTSS is evaluated by using Cronbach’s α and retest reliability. All data in this study do not contain missing data.

## Results

### Item analysis

First, we select the volunteers with the highest 27% of the total scores as the high-score group and those with the lowest 27% as the low-score group. Then *t*-test is performed to examine the differences between the high- and low-score groups for each item, exhibiting a significant difference (*Ps* < 0.001) between both groups. In addition, we also calculate the item-total correlation of each item and find that all the items meet the criterion of 0.3 (Nunnally & Bernstein, 1994). The item-to-total correlation ranges from 0.77 to 0.84 (*Ps* < 0.001). Therefore, all items of the scale are retained. The distributional properties of most TTSS items present satisfactory univariate normality, as shown in Table 1.

**Table 1:** Factor loading, communality, mean, standard deviation, skewness, kurtosis, and item-total correlation of TTSS (*n* = 603)

Item	Positive social	Curiosity/stress	Communality	*M*	*SD*	Skewness	Kurtosis	Item-total correlation
TTSS				8.85	3.66	2.48	3.55	
1. While talking to my friends	0.91		0.85	1.30	0.67	3.24	4.50	0.78***
2. When I am having a good time	0.87		0.76	1.42	0.73	2.13	3.66	0.77***
3. When I want to be part of the crowd	0.82		0.79	1.44	0.76	2.26	3.29	0.79***
4. When others are talking about how much they like smoking		0.89	0.86	1.43	0.78	2.15	2.94	0.84***
5. When I am stressed		0.84	0.80	1.58	0.82	1.57	1.25	0.84***
6. When I want to know how a cigarette tastes		0.82	0.87	1.46	0.80	1.79	1.70	0.80***
Eigenvalue	3.88	1.05						
Contribution		82.19%						

^***^Means *P* < 0.001, which is the same for the following items

### Construct validity

#### Exploratory factor analysis (EFA)

The Bartlett test of sphericity shows that KMO (the Kaiser–Meyer–Olkin) = 0.83, *χ*^2^ = 1247.73 (*df* = 15), and *P* < 0.001, indicating that the data is suitable for EFA (Kaiser, 1974). Using principal component analysis and the varimax rotation method, two factors are generated based on the criterion of an eigenvalue greater than 1, with 82.19% of the total variance accounted. Finally, the six-item Chinese TTSS is obtained. According to the meanings of the items, the two factors are labeled as positive social and curiosity/stress. The factor loading of each item ranges from 0.82 to 0.91, and the communality ranges from 0.76 to 0.87, as shown in Table 1.

#### Confirmatory factor analysis (CFA)

**Fig. 1:**
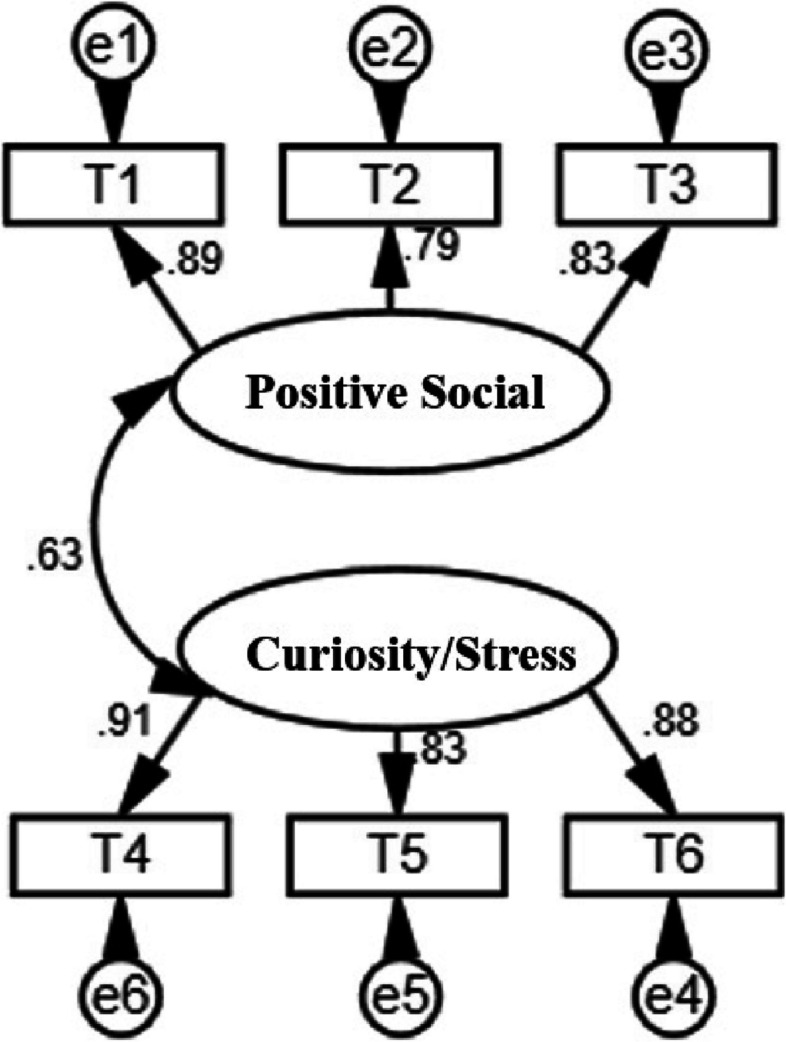
Diagram of the Chinese version of the TTSS two-factor structural equation model

The maximum likelihood estimation is used for CFA, and the standardized factor loading is valued between 0.79 and 0.91. The fit index of the two-factor structure model is good (Bentler, 1990) (Fig. 1), whereas the single-factor model does not meet the measurement standard (Table 2).

**Table 2:** Fit index of the model (*n* = 592)

Model	*χ*^2^	*df*	*χ*^2^/*df*	RMR	*CFI*	*GFI*	*TLI*	*IFI*	*RMSEA*
Two factor	14.11	6	2.35	0.02	0.99	0.97	0.98	0.98	0.06
Single factor	56.89	9	6.32	0.07	0.85	0.85	0.69	0.86	0.13

*RMR* root-mean-square residual, *CFI* comparative fit index, *RFI* relative fit index, *GFI* goodness-of-fit index, *TLI* Tucker-Lewis index, *IFI* incremental fit index, *RMSEA* root-mean-square error of approximation

### Criterion-related validity

The TTSS is positively correlated with positive social and curiosity/stress, while positive social is positively correlated with curiosity/stress. The total scores of TTSS, positive social, and curiosity/stress are positively correlated with the scores of Pros, ASCOS, TAS, and Dis of SSS and negatively correlated with Cons (Table 3).

**Table 3:** Correlations between TTSS scores and ASCOS, CDBS, and SSS scores (*n* = 592)

	TTSS	Positive social	Curiosity/stress	Pros	Cons	ASCOS	TAS	Dis
TTSS	1							
Positive social	0.87***	1						
Curiosity/stress	0.90***	0.58***	1					
Pros	0.42***	0.39***	0.36***	1				
Cons	− 0.35***	− 0.29***	− 0.33***	− 0.22***	1			
ASCOS	0.37***	0.26***	0.39***	0.23***	− 0.22***	1		
TAS	0.24***	0.16***	0.27***	0.23***	− 0.15***	0.25***	1	
Dis	0.46***	0.34***	0.26***	0.32***	− 0.22***	0.29***	0.46***	1

*TAS* thrill and adventure seeking, *Dis* disinhibition

*Note*: ***Means *P* < 0.001

### Reliability

#### Internal consistency

Cronbach’s *α* is used to evaluate the internal consistency of the scale. The results show that Cronbach’s *α* of the TTSS is 0.89, and the positive social and curiosity/stress are 0.87 and 0.90, respectively.

#### Test–retest reliability

The correlation analysis shows that the test–retest reliability (*r*) of the TTSS is 0.90 (*P* < 0.001), and the positive social and curiosity/stress are 0.88 and 0.89, respectively. These results show that all these tests are valid in statistics (*Ps* < 0.001).

## Discussion

Currently, research on the prevention and intervention of smoking attempts in China is still insufficient because few measurement tools are available for adolescents. Considering that adolescents are located in a sensitive period of physical and mental development, they are not able to resist temptation and are more likely to try smoking under their environmental temptation. In addition, adolescents who try smoking are likely to become lifelong smokers. Therefore, it is necessary to compile or revise a measurement tool in China for adolescents’ temptation to try smoking, which can also provide an assessment tool for effective prevention and intervention. This study strictly follows the standardized translation process, develops the Chinese version of the TTSS, and tests its reliability and validity.

Using the discrimination and correlation method to analyze the items of the scale, the critical values of all the 6 items are extremely significant (*Ps* < 0.001). The correlation between items and the total scale ranges from 0.77 to 0.84, indicating that the TTSS has good discrimination. Therefore, all 6 items should be retained. The EFA yields two factors of positive social and curiosity/stress, which are consistent with the two-factor structure of the original English version. The CFA results show that the fit index of the two factors is good, which reaches good psychometric standards. It indicates that the Chinese version of the TTSS has good construct validity. The Chinese version of the TTSS has the same structure with the English version, indicating that the 6 items have measured the level of temptation to try smoking in Chinese adolescents from different aspects. It proves that the TTSS has cross-cultural consistency.

The Chinese version of the TTSS also has good criterion-related validity. In this study, CDBS, ASCOS, and SSS are used as the criteria. The achieved results show that the total scores of the TTSS, positive social, and curiosity/stress are positively correlated with the scores of Pros, ASCOS, TAS, and Dis of SSS and negatively correlated with the Cons. This result is consistent with previous studies (Crawford et al., 2003) and also shows a similar conclusion in the TTM (Prochaska & Velicer, 1997; Velicer et al., 2000). According to the four components of TTM (stage of change, process of change, decisional balance, and situational temptations), the temptation to try smoking, as a kind of situational temptation, reflects an individual’s strong desire to smoke in a specific situation. It affects an individual’s self-efficacy and is the best predictor of relapse and regression of behavior, so it plays a significant role in the prediction of behavior change (Plummer et al., 2001).

The reliability test reveals that Cronbach’s α of the TTSS is 0.89, and Cronbach’s α of positive social and curiosity/stress are 0.87 and 0.90, respectively. They are consistent with the original English version. After 1 month, the test–retest reliability of the TTSS is 0.90 with the positive social score of 0.88 and curiosity/stress score of 0.89. This result obeys the psychometric criteria, which means that the Chinese version of the TTSS has good internal consistency and stability.

The two-factor structure of the TTSS tested among Chinese adolescents is consistent with the structure presented in previous work (McGee et al., 2012). All the indicators meet the psychometric standards, exhibiting good reliability and validity. TTSS can not only be used to assess the level of temptation to try smoking in Chinese adolescents but also suggest a scientific evaluation tool for the consequent prevention and intervention of adolescent smoking behavior.

We translate the TTSS into a Chinese scale and conduct a rigorous psychometric assessment. Overall, the results suggest that the TTSS is an effective tool to measure changes in smoking behavior among Chinese adolescents. Based on the theory of the TTM, the development of scale is considered an indispensable prerequisite for any attempt to implement and evaluate health education and to promote prevention and intervention (Sarbandi et al., 2013). “Temptation,” as a central theme in addiction literature, the Chinese version of the Temptations to Try Smoking Scale, can make a valuable contribution to the change in smoking behavior among Chinese adolescents. Thus, the results of this study can be well applied to the prevention and intervention of smoking attempts among Chinese adolescents.

Several limitations should be highlighted. First of all, the volunteers are middle school students from only four provinces in China, which may not be fully representative of the entire Chinese youth group. Secondly, the volunteers are not recruited with a random sampling, so the generality of the results might be limited. Thirdly, the obtained data may be affected by personal bias, although the survey is anonymous. Despite these limitations, the present study provides solid evidence of the psychometric properties of TTSS in Chinese adolescents.

## Conclusion

This study has examined the factor structure, reliability, and standard correlation validity of TTSS in Chinese adolescents. The results indicate that the TTSS has good psychometric properties in China. Particularly, the Chinese TTSS has a clear factor structure with an acceptable fit index. The Cronbach’s α coefficient and retest reliability of the Chinese TTSS are high. The total scores of TTSS and the scores of positive social and curiosity/stress subscales are significantly positively correlated with the scores of Pros of CDBS, ASCOS, and SSS (both TAS and Dis) but negatively correlated with the scores of Cons of CDBS. It indicates that Chinese TTSS has high criterion correlation validity, suggesting that the Chinese version of TTSS is a valid and reliable tool to assess Chinese adolescents’ temptation to try smoking.

## Data Availability

The data that support the findings of this study are available on request from the corresponding author. The data are not publicly available due to privacy or ethical restrictions.

## Abbreviations

TTM: The transtheoretical model
TTSS: Temptations to Try Smoking Scale
CDBS: The Chinese version of the Decisional Balance Scale
ASCOS: Adolescent Smoking Curiosity Scale
SSS: Sensation-Seeking Scale
TAS: Thrill and adventure seeking
Dis: Disinhibition
EFA: Exploratory factor analysis
CFA: Confirmatory factor analysis
KMO: Kaiser-Meyer-Olkin
RMR: Root-mean-square residual
CFI: Comparative fit index
RFI: Relative fit index
GFI: Goodness-of-fit index
TLI: Tucker-Lewis index
IFI: Incremental fit index
RMSEA: Root-mean-square error of approximation
